# Ponicidin Inhibits Lung Cancer Progression Through Coordinated Downregulation of Sulfhydryl Antioxidants and TrxR1

**DOI:** 10.3390/antiox15010100

**Published:** 2026-01-13

**Authors:** Yufei Huang, Yanfen Liu, Zehua Liao, Ruonan Zhang, Xinbing Sui, Xueni Sun

**Affiliations:** School of Pharmacy, Hangzhou Normal University, Hangzhou 311121, China

**Keywords:** ponicidin, lung cancer, oxidative stress, sulfhydryl antioxidants, TrxR1

## Abstract

Ponicidin, a bioactive diterpenoid derived from *Rabdosia rubescens*, has been shown to exhibit antitumor activity across a range of cancer types. Despite its potential therapeutic applications, the precise effects and underlying molecular mechanisms of ponicidin in the context of lung cancer remain insufficiently characterized. This study aims to investigate the antitumor effects of ponicidin in lung cancer, focusing on its impact on cell growth and cellular oxidative stress. Our findings demonstrate that ponicidin significantly inhibits the viability of lung cancer cells while exhibiting minimal cytotoxicity to normal lung cells. Notably, ponicidin induces cell death in lung cancer cells via the induction of oxidative stress, a process likely mediated by the depletion of sulfhydryl antioxidants and the downregulation of thioredoxin reductase (TrxR), both of which play critical roles in maintaining cellular redox homeostasis. Moreover, ponicidin was found to concurrently activate endoplasmic reticulum stress, induce mitochondrial dysfunction, and promote DNA damage, further contributing to its antitumor effects. In vivo, the efficacy of ponicidin was confirmed in tumor-bearing mouse models, where ponicidin treatment led to a significant reduction in tumor growth without significant toxicity or adverse effects on the animals. These findings suggest that ponicidin holds significant promise as a safe and effective therapeutic agent for lung cancer, warranting further investigation into its clinical applicability.

## 1. Introduction

Globally, lung cancer is the primary cause of cancer-related deaths [1]. Lung adenocarcinoma and lung squamous cell carcinoma are the two most prevalent histological subtypes of non-small-cell lung cancer (NSCLC), which affects about 85% of patients [2]. Currently used therapeutic approaches include surgical resection [3], chemotherapy [4], targeted therapies [5], and immunotherapies [6]. However, conventional chemotherapeutic agents face significant limitations, including nonspecific targeting [7], low bioavailability [8], and the emergence of medication resistance [9], which limit their efficacy in cancer treatment. Consequently, the prognosis of lung cancer remains considerably poor [10]. Traditional Chinese medicine (TCM) has been investigated for its potential complementary role in cancer treatment, including NSCLC, due to its unique advantages in symptom relief [11], prolonged survival [12], immune function modulation [13], and quality of life improvement [14].

*Rabdosia rubescens* (Hemsl.) Hara, commonly referred to as Dong-ling-cao in China, is a prominent Chinese medicinal herb renowned for its therapeutic properties in TCM. It is extensively utilized for its documented pharmacological effects, notably recognized for its antitumor [15] and anti-inflammatory [16] activities. This herb contains numerous chemical constituents, including terpenoids, volatile oils, alkaloids, flavonoids, and polysaccharides. These include ent-kaurane diterpenoids like ponicidin and oridonin, are the primary active constituents with significant potential in cancer therapy [17,18]. Among them, oridonin has been extensively investigated. It is a promising anticancer adjuvant, demonstrated to selectively induce apoptosis and cell cycle arrest in cancer cells with minimal cytotoxicity to normal cells [19]. Ponicidin, its structural analog, has also garnered attention for its broad-spectrum anticancer activities. Preclinical studies have established that ponicidin inhibits proliferation, induces apoptosis, and suppresses metastasis in various cancers, including hepatocellular carcinoma, gallbladder cancer, and esophageal squamous cell carcinoma [20,21,22,23,24]. Mechanistic explorations suggest its actions involve modulating key pathways such as SLC7A11/Glutathione, CHPF2, and KEAP1/Nrf2 signaling. However, despite this foundational evidence of its bioactivity, research focusing specifically on lung cancer is strikingly scarce. The existing data do not adequately address whether and how ponicidin exerts antitumor effects against lung cancer cells, nor do they clarify its potential to overcome therapy resistance in its malignancy. Consequently, the precise mechanism of action of ponicidin within the context of lung cancer pathogenesis remains largely unelucidated, representing a critical gas that this study aims to address.

Human health depends on maintaining redox equilibrium, and many diseases, including cancer, are linked to its disturbance [25]. Oxidative stress, characterized by an imbalance in redox signaling, is commonly observed in cancer cells. Consequently, therapeutic strategies that exploit oxidative stress vulnerability have emerged. Reactive oxygen species (ROS) are natural by-products of cellular processes such as mitochondrial metabolism and protein folding [26]. While ROS play roles in cellular signaling, their excess is normally neutralized by the antioxidant system, which includes enzymes like peroxiredoxins (PRDXs), superoxide dismutases (SOD), and thioredoxin reductases (TrxR) [27]. Notably, these antioxidant proteins are often upregulated in cancer cells to mitigate ROS levels. However, when ROS production surpasses the antioxidant capacity, it leads to cellular damage through pathways involving DNA damage and endoplasmic reticulum (ER) stress, ultimately inducing cell death [28]. Many conventional chemotherapeutic agents, including paclitaxel [29], cisplatin [30], 5-fluorouracil [31], and arsenic trioxide [32], cause the death of cancer cells by either directly or indirectly encouraging the formation of ROS. Based on these insights, pharmaceuticals capable of modulating cellular oxidative homeostasis represent a promising way for advancing cancer therapy.

This study examines the inhibitory impact of ponicidin on lung cancer using in vitro and in vivo models, aiming to clarify its potential mechanism of action. These results demonstrate that ponicidin induces cell death in lung cancer cells by increasing oxidative stress, as evidenced by elevated levels of ROS. This effect coincides with observable damage to mitochondria, ER, and DNA molecules. These findings aim to establish a basis for understanding the potential development and mechanisms of ponicidin as a novel natural therapeutic agent against lung cancer through the modulation of redox homeostasis.

## 2. Materials and Methods

### 2.1. Chemicals

The supplier of ponicidin (purity > 98%, B20311) was Shanghai Yuanye Bio-Technology Co., Ltd. (Shanghai, China). MCE provided the following: mitoquinone mesylate (HY-100116A), acetylcysteine (HY-B0215), L-glutathione reduced (HY-D0187), and naph-EA-mal (HY-D1261). Antibodies targeting PRDX1 (#8499) and GAPDH (#5174), and TrxR1 (#15140) were sourced from Cell Signaling Technology (Shanghai, China), while ABclonal (Wuhan, China) provided the antibodies against Trx1 (A7638).

### 2.2. Cell Culture

NSCLC cell lines NCI-H1975 (H1975) and NCI-H1299 (H1299) were obtained from the National Collection of Authenticated Cell Cultures (Shanghai, China) and Cell Resource Center, Institute of Basic Medical Sciences (Beijing, China), respectively. All cell lines were authenticated upon receipt using STR profiling and were confirmed to be mycoplasma-free. H1299 and H1975 cells were cultivated in RPMI-1640 media. Every cell line was cultured at 37 °C in an atmosphere with 5% CO_2_.

### 2.3. Cell Viability Assay

We used a cell counting kit-8 (CCK-8, Meilunbio) to evaluate each cell line’s viability. Cell viability (%) = [A(experiment) − A(blank)]/[A(control) − A(blank)] × 100%, where A(experiment) is the absorbance of the treated wells, A(blank) represents the absorbance of wells containing medium and CCK-8 reagent without cells, and A(control) is the absorbance of the untreated control wells (cells with CCK-8 reagent but no drug treatment).

### 2.4. Cell Staining

The Calcein/PI Cell Activity and Cytotoxicity Assay Kit (C2015S) were acquired from Beyotime (Shanghai, China) for evaluating the impact of ponicidin on lung cancer cell activity. Calcein-AM and propidium iodide (PI) were employed to assess intracellular esterase activity and cell membrane integrity, respectively, thereby indicating cell activity and cytotoxicity. Following the manufacturer’s protocol, cells were stained and imaged using an inverted fluorescence microscope. For Calcein (the hydrolyzed product of Calcein-AM), images were captured at its peak excitation/emission wavelengths of 494 nm/517 nm. For PI, images were captured at its peak excitation/emission wavelengths of 535 nm/617 nm. All images for a given experiment were acquired using identical exposure times and camera gain settings to allow for comparative analysis.

### 2.5. Colony Formation Assay

Seeded into 10 cm dishes, 8 × 10^3^ cells per well were incubated at 37 °C with 5% CO_2_. Following seeding, the cells underwent individual treatments for a whole day using varying doses of ponicidin. Following the replacement of the culture medium, colony formation was periodically observed under a microscope at intervals of three days. The clonal spheres were photographed for further analysis after they reached a diameter of 0.5–1 mm, were dyed with a 0.1% crystal violet solution, and were fixed with a 4% fixative solution. Image J software (version 1.53) was then used to count the monoclones.

### 2.6. Cell Apoptosis Analysis

Cell apoptosis was investigated using Annexin V/PI staining from the apoptosis detection kit (40302ES60), purchased from Yeasen Biotechnology Co., Ltd. (Shanghai, China). 2 × 10^5^ cells per well were seeded onto 6-well plates for this assay, and they were left to incubate in a humidified incubator for the whole night. After treatment with ponicidin, cells were harvested, stained according to the manufacturer’s instructions, and analyzed by flow cytometry (CytoFLEX S, Beckman Coulter, Brea, CA, USA). For each sample, 10,000 events were collected for quantitative analysis.

### 2.7. ROS Assay

Using 2′,7′-dichlorodihydrofluorescein diacetate (DCFH-DA) from Beyotime (S0033M), the intracellular build-up of ROS, a marker of oxidative stress, was measured. First, the cells were treated with DCFH-DA in serum-free medium for 30 min at 37 °C. Following cell harvesting, a flow cytometer (CytoFLEX S, Beckman Coulter) was used to measure the green fluorescence released by oxidized DCFH-DA.

### 2.8. RT-qPCR

TRIzol (Invitrogen, Carlsbad, CA, USA) was used to extract total RNA, and the TransScript All-in-One First-Strand cDNA Synthesis SuperMix for qPCR (One-Step gDNA Removal) (AT341-03, TransGen Biotech, Beijing, China) was used to reverse-transcribe the RNA to complementary DNA (cDNA). mRNA expression was examined using TransGen Biotech’s PerfectStart Green qPCR SuperMix (+Dye II) (AQ602-23). All of the primer sequences utilized in the study are included in Table 1. The expression of the GAPDH gene serves as a control for normalization.

### 2.9. Western Blot Analysis

RIPA buffer (Beyotime, P0013B) was used to collect the cells (Beyotime, ST506). The BCA Protein Assay Kit (Beyotime, P0012S) was used to measure the protein concentrations. Equal amounts of total protein (20 μg per lane) were denatured, separated by SDS-PAGE and transferred onto PVDF membranes. After that, the membranes were incubated with the designated primary antibodies for an entire night. They were then subjected for two hours to secondary antibodies. ChemiDoc equipment (Bio-Rad, Hercules, CA, USA) was used to take band pictures, and Image J software was used for analysis.

### 2.10. Mitochondrial Membrane Potential Assay

The mitochondrial membrane potential assay kit with JC-1 probe was used to measure the mitochondrial membrane potential (Beyotime, C2006). Mitochondrial depolarization was shown by the proportion of red to green fluorescence. Flow cytometry and fluorescence microscopy were used for quantitative or qualitative analysis.

### 2.11. ER Tracker Assay and Ca^2+^ Concentration Detection

The Endoplasmic Reticulum tracker (ER tracker-green, Beyotime, C1042S) was used for ER localization. In short, cells were incubated for 30 min at 37 °C in an atmosphere with 5% CO_2_ after being exposed to a preheated ER tracker dye working solution. After being incubated, the nuclei were then stained with Hoechst 33,342 (Beyotime, C1022) for ten minutes at room temperature. Finally, an inverted fluorescence microscope was used to observe and image the cells. Intracellular free Ca^2+^ levels were detected using Fluo-4 AM (Beyotime, China, S1061S). To put it briefly, the cells were treated at 37 °C for 30 min with Fluo-4 AM. The cells were imaged under an inverted fluorescent microscope.

### 2.12. Immunofluorescence Staining for γ-H_2_AX

Immunofluorescence was utilized to examine the expression patterns of γ-H_2_AX in cells. Briefly, cells were seeded onto coverslips, fixed with 4% methanol for 15 min, and then incubated at 4 °C for the entire night with an anti-γ-H_2_AX primary antibody. DAPI (SL7101, Coolaber, Beijing, China) was used to counterstain the nuclei after the cells had been treated with the secondary antibody (Goat Anti-Rabbit IgG H&L). Following that, a Confocal Microscope (Olympus, model FV3000, Tokyo, Japan) was used to image and study the cells.

### 2.13. In Vivo Experiments

Every animal experiment was carried out in compliance with Hangzhou Normal University’s Use and Care of Animals Committee’s regulations. The BALB/c nude mice were acclimated for one week before receiving a subcutaneous injection of 100 μL of H1975 cells suspended in phosphate-buffered saline (5 × 10^6^ cells/mL) into their right flank for in vivo tumor growth studies. Ten days post-injection, mice were randomly assigned into three experimental groups (*n* = 5 mice per group) using a random number sequence and given either 0 mg/kg ponicidin, 20 mg/kg ponicidin, or 30 mg/kg ponicidin. Throughout the experiment, investigators involved in tumor measurement and body weight recording were blinded to the group allocations. Drug solutions were prepared and coded by a separate researcher. In order to test the theory that ponicidin prevents lung cancer by causing oxidative stress in vivo, we built the H1299 animal model. One week after acclimatization, BALB/c naked mice were given a subcutaneous injection of 100 μL of H1299 cells suspended in PBS (5 × 10^6^ cells/mL) into their right abdomen. The mice were divided into five groups at random (*n* = 5 per group) two weeks following injection: blank control group, ponicidin treatment group (30 mg/kg), NAC group (100 mg/kg), ponicidin + NAC group, and cisplatin positive control group (2 mg/kg). Following the initial medication treatment, body weight and tumor long and short diameters were measured every two days. At the end of the experiment, orbital blood sampling was conducted on nude mice, and blood samples were processed to assess the levels of ALT, AST, Urea, CR, and other relevant indexes, using a blood cell analyzer. The experimental procedures involving animals were reviewed and approved by the Animal Ethics and Welfare Committee (AEWC) of Hangzhou Normal University (Approval Code: HSD-20250428-04; Date of Approval: 29 April 2025).

### 2.14. Statistical Analysis

All of the data were analyzed using GraphPad Prism 8.0. Data from at least three independent biological replicates (*n* ≥ 3) are presented as mean ± SD. After normality assessment, the *t*-test was used to evaluate differences between two groups, and a one-way or two-way ANOVA was employed for comparisons involving multiple groups, followed by post hoc multiple comparisons tests. Dunnett’s test was selected for comparisons where all experimental groups were compared against a single control group, while Tukey’s test was employed for all pairwise comparisons between groups. P was considered statistically significant when it was less than 0.05.

## 3. Results

### 3.1. Ponicidin Suppresses Lung Cancer Cell Proliferation and Induces Cell Death

**Figure 1:**
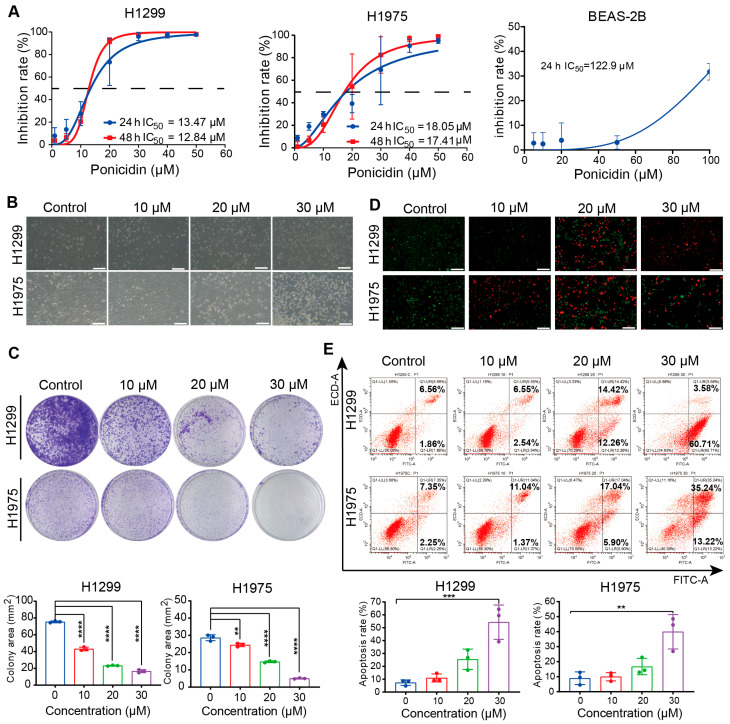
Ponicidin suppresses lung cancer cell growth and induces cell death. (**A**) H1299, H1975, and BEAS-2B cells were treated with different concentrations of ponicidin for 24 h and 48 h, and cell viability was measured by CCK-8 assay, mean ± SD, *n* = 3. The dashed line marks the point of 50% inhibition. (**B**) The cells morphology change was monitored under a microscope after the treatment with ponicidin for 24 h, scale bar = 200 μm. (**C**) Representative results of the colony formation and quantitative analysis, mean ± SD, *n* = 3, ** *p* < 0.01, **** *p* < 0.0001. (**D**) The live and dead cells were stained using the LIVE/DEAD Viability/Cytotoxicity Kit (Cells stained with green fluorescence are live cells labeled by Calcein, while those stained with red fluorescence are dead cells labeled by PI), scale bar = 200 μm. (**E**) Representative results of annexin V-FITC/PI staining and quantitative analysis after the treatment with ponicidin for 24 h, mean ± SD, *n* = 3, ** *p* < 0.01, *** *p* < 0.001.

The pharmacotoxicity profile and anticancer potential of ponicidin were comprehensively assessed in human normal lung epithelial cells (BEAS-2B) and lung cancer cell lines (H1299 and H1975). Both H1299 and H1975 lung cancer cell lines showed a concentration-dependent decrease of cell viability after being treated with different doses of ponicidin for 24 and 48 h. The calculated IC_50_ values for ponicidin treatment were 13.47 μM (24 h, H1299), 18.05 μM (24 h, H1975), 12.84 μM (48 h, H1299), and 17.41 μM (48 h, H1975) (Figure 1A). Notably, the normal lung epithelial cell line BEAS-2B showed few harmful effects. The IC_50_ values at the 24 h time point for BEAS-2B cells (122.9 μM) were significantly higher than those for the lung cancer cells, by factors of 9.12 and 6.81, respectively, underscoring the selective cytotoxicity of ponicidin toward malignant lung cancer cells.

Optical microscopy further revealed notable alterations in cellular morphology upon ponicidin treatment, including increased intercellular spacing and the presence of floating cells at higher concentrations of ponicidin (Figure 1B). These morphological changes were consistent with the observed suppression of cell proliferation. In addition, the clonogenic assay demonstrated a dose-dependent reduction in colony formation in ponicidin-treated lung cancer cells, compared to control groups, further corroborating its inhibitory effect on cellular proliferation (Figure 1C).

Additionally, cell viability was assessed using fluorescence-based viability staining, which showed an increase in red fluorescence intensity corresponding to elevated concentrations of ponicidin (Figure 1D). This increase in fluorescence intensity was indicative of reduced cell viability and enhanced cell death in ponicidin-treated lung cancer cells. However, at a ponicidin concentration of 30 μM, an artifact was observed during the experimental procedure: the accidental removal of dead cells during supernatant suction led to a drop in the overall cell count, and subsequently a fall in red fluorescence intensity in the experimental data. Furthermore, flow cytometric examination employing Annexin V-FITC/PI labeling demonstrated a substantial increase in the percentage of death cells following ponicidin treatment, compared to the untreated controls (Figure 1E). These findings provide strong evidence for the inhibitory effect of ponicidin on cell proliferation and its capacity to induce cell death in lung cancer cells.

### 3.2. Ponicidin Inhibits Lung Cancer Cell Growth In Vivo

**Figure 2:**
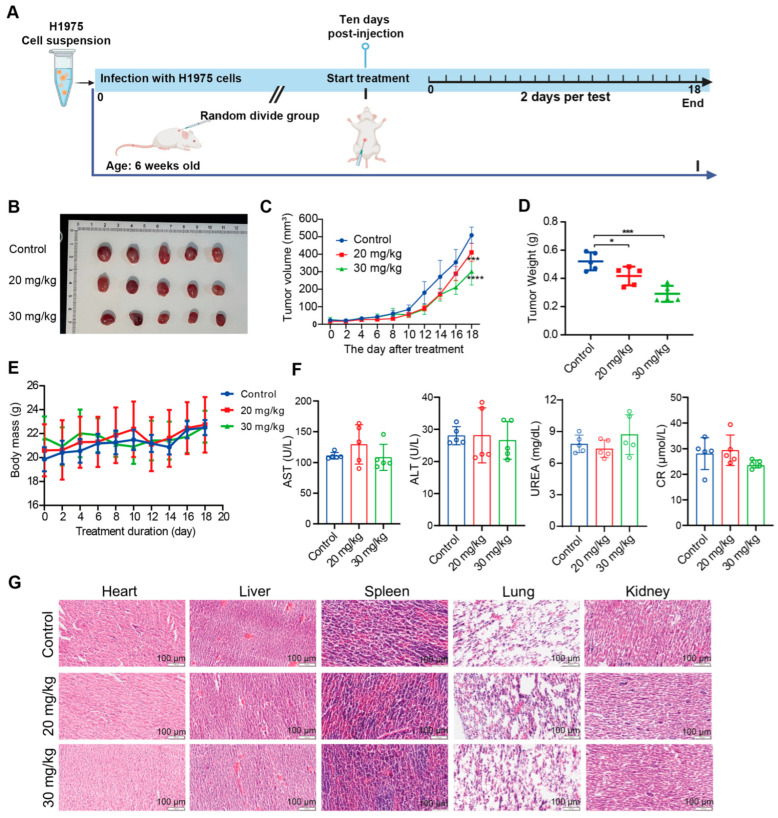
Ponicidin suppresses lung cancer cell growth in vivo. (**A**) H1975 cells were subcutaneously injected into the backs of nude mice, and mice were intraperitoneally injected with 0 mg/kg ponicidin (solvent control), 20 mg/kg ponicidin, and 30 mg/kg ponicidin. (**B**) Mice were euthanized after 18 days’ treatment, and tumors were extracted and photographed. (**C**) Changes of tumor volumes in each group were monitored, mean  ±  SD, *n* = 5, *** *p* < 0.001, **** *p* < 0.0001. (**D**) Tumor weights were evaluated, mean  ±  SD, *n* = 5, * *p* < 0.05, *** *p* < 0.001. (**E**) Changes of body weights of mice in each group were monitored, mean  ±  SD, *n* = 5. (**F**) Blood samples were collected for biochemical analysis, mean  ±  SD, *n* = 5. (**G**) Heart, liver, spleen, lungs, and kidneys were collected for histological examination via H&E staining, scale bar = 100 μm.

To validate our in vitro observations, we extended our investigation to assess the effects of ponicidin in an in vivo xenograft model using nude mice. H1975 lung cancer cells were subcutaneously inoculated into the mice. The animals were randomly assigned into four groups (*n* = 5 per group): a solvent control group, a low-dose ponicidin group (20 mg/kg), and a high-dose ponicidin group (30 mg/kg) (Figure 2A). As expected, both the low and high doses of ponicidin significantly suppressed tumor growth compared to the control group (Figure 2B–D). Interestingly, while no notable differences were observed between the low- and high-dose groups during the early phase of treatment, clear distinctions between the two groups emerged in the later stages. By the end of the study, both the low- and high-dose ponicidin groups exhibited substantial reductions in tumor volume compared to the control (Figure 2C), and the tumor weights in these groups were significantly lower than those of the control group (Figure 2D). Throughout the duration of the experiment, the body weight of the mice in all groups showed a slight increase, with no discernible differences observed among the groups (Figure 2E), indicating the overall health of the animals under ponicidin treatment.

To further assess the potential toxic effects of ponicidin on systemic organs, we conducted biochemical analysis of blood samples, measuring key biomarkers including AST, ALT, blood urea, and creatinine (CR), which are commonly used to assess liver and kidney function. The results showed no discernible changes in the liver or kidney function of ponicidin-treated mice compared to the control group (Figure 2F). In order to identify any possible pathological alterations, a histological examination of the main organs, the heart, liver, spleen, lungs, and kidneys, was also carried out using H&E staining. When comparing the mice treated with ponicidin at both doses to the control group, the histological study revealed no discernible pathological damage in any of the organs (Figure 2G). These findings collectively indicate that ponicidin effectively inhibits the growth of lung cancer cells in vivo without causing detectable toxic effects on liver, kidney, or other major organ functions in nude mice.

### 3.3. Ponicidin Increases ROS Level in Lung Cancer Cells

**Figure 3:**
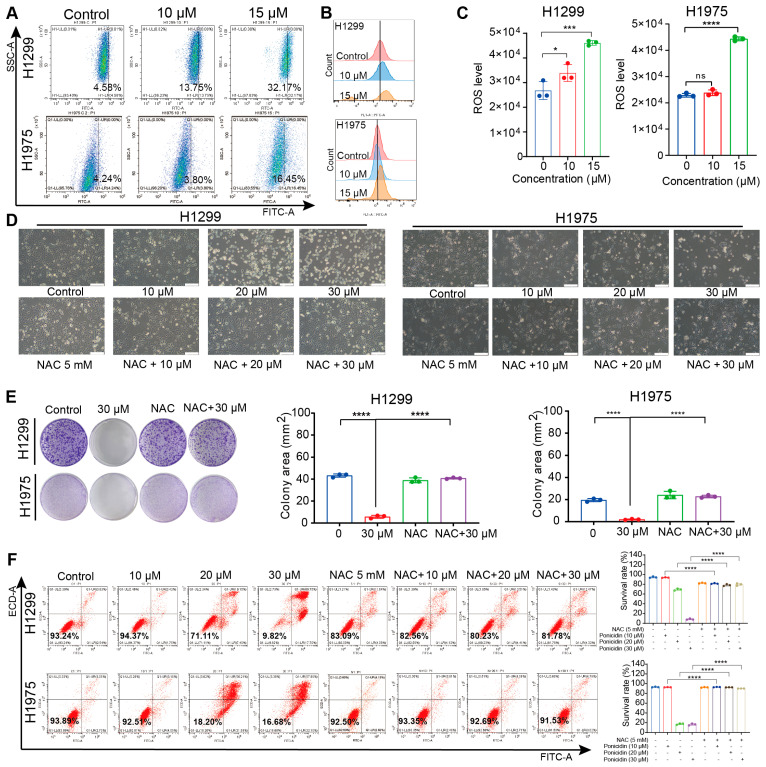
Ponicidin increases intracellular ROS level in lung cancer cells and NAC antagonizes the inhibitory effect of ponicidin on lung cancer cells. (**A**–**C**) H1299 and H1975 cells were treated with ponicidin for 24 h and stained with 10 μM DCFH-DA. ROS levels were analyzed by flow cytometer and the bar charts display the results of quantified analysis, mean ± SD, *n* = 3, * *p* < 0.05, *** *p* < 0.001, **** *p* < 0.0001, ns: no significance. (**D**) H1299 and H1975 cells were pretreated with NAC for 2 h followed by treatment with ponicidin for 24 h, and the cells morphology change was detected under a microscope, scale bar = 200 μm. (**E**) H1299 and H1975 cells were pretreated with NAC for 2 h followed by treatment with ponicidin for 24 h, and representative results of the colony formation and quantitative analysis, mean ± SD, *n* = 3, **** *p* < 0.0001. (**F**) Representative results of annexin V-FITC/PI staining and quantitative analysis after cells were pretreated with NAC for 2 h followed by treatment with ponicidin for 24 h, mean ± SD, *n* = 3, **** *p* < 0.0001, ns: no significance; “-”: without the corresponding treatment; “+”: with the corresponding treatment.

Given the well-established association between elevated ROS levels and the induction of cell death in various cancer types, we sought to investigate the impact of ponicidin treatment on ROS production in lung cancer cells. As expected, our results demonstrated a significant increase in ROS levels in both H1299 and H1975 lung cancer cell lines following ponicidin treatment (Figure 3A–C), indicating a disruption in intracellular ROS homeostasis. To further substantiate the role of ROS in ponicidin-induced cytotoxicity, we performed a rescue experiment using N-acetylcysteine (NAC), a well-known ROS scavenger. Pretreatment with NAC, followed by co-administration with ponicidin, significantly mitigated the morphological alterations and growth inhibition induced by ponicidin, restoring cell morphology and proliferation to levels comparable to the control group (Figure 3D). Similarly, the exogenous addition of NAC effectively reversed the ponicidin-induced reduction in colony formation (Figure 3E) and attenuated the cell death observed in ponicidin-treated lung cancer cells (Figure 3F). These findings suggest that the elevated ROS levels induced by ponicidin are likely central to its mechanism of action in promoting cell death in lung cancer cells, highlighting the potential of targeting oxidative stress pathways for cancer therapy.

### 3.4. Ponicidin Induces the Reduction of Sulfhydryl Compounds and Depletion of Glutathione, Thereby Regulating Oxidative Stress in Lung Cancer Cells

**Figure 4:**
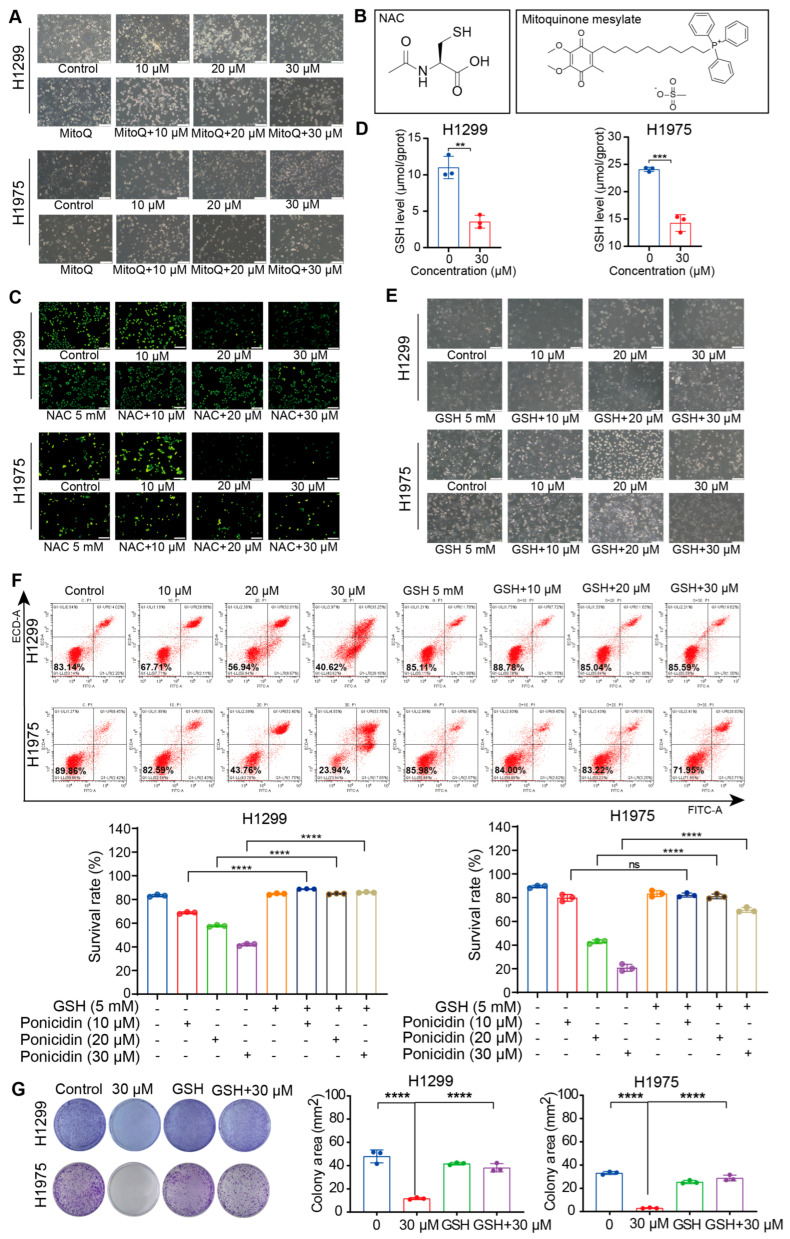
Ponicidin induced reduction of sulfhydryl compounds and glutathione depletion, regulating oxidative stress levels in lung cancer cells. (**A**) H1299 and H1975 cells were pre-treated with Mitoquinone mesylate (2 μM) for 2 h followed by co-incubation with various concentrations of ponicidin for 24 h, and the cells morphology change was detected under a microscope, scale bar = 200 μm. (**B**) The chemical structure of NAC and Mitoquinone mesylate. (**C**) H1299 and H1975 cells were treated with ponicidin for 24 h, with or without NAC pretreatment, then the cells were stained with Naph-EA-mal fluorescent dye to detect changes in sulfhydryl group content in living cells, scale bar = 200 μm. (**D**) Ponicidin reduced intracellular GSH levels in lung cancer cells. The GSH levels were measured by GSH Assay Kit and normalized to protein level, mean ± SD, *n* = 3, ** *p* < 0.01, *** *p* < 0.001. (**E**) H1299 and H1975 cells were pre-treated with GSH for 2 h followed by co-incubation with various concentrations of ponicidin for 24 h, and the cells morphology change was detected under a microscope, scale bar = 200 μm. (**F**) Representative results of annexin V-FITC/PI staining and quantitative analysis after GSH co-administration with various concentrations of ponicidin for 24 h, mean ± SD, *n* = 3, **** *p* < 0.0001, ns: no significance; “-”: without the corresponding treatment; “+”: with the corresponding treatment. (**G**) H1299 and H1975 cells were pre-treated with GSH for 2 h followed by co-incubation with various concentrations of ponicidin for 24 h, and representative results of the colony formation and quantitative analysis, mean ± SD, *n* = 3, **** *p* < 0.0001.

To further investigate the role of ROS in ponicidin-induced cell death, we assessed the efficacy of another ROS scavenger, Mitoquinone mesylate, a mitochondria-targeted antioxidant. H1299 and H1975 cells were pretreated with Mitoquinone mesylate (2 μM) for 2 h, followed by co-incubation with various concentrations of ponicidin for 24 h. Surprisingly, Mitoquinone mesylate did not effectively rescue ponicidin-induced cell death in lung cancer cells (Figure 4A). This suggests that ponicidin-induced cell death may not solely rely on ROS induction, and the mechanism by which NAC exerted its protective effects in previous experiments may involve other factors. To dig deeper into this, we compared the chemical structures of these two ROS scavengers, NAC and Mitoquinone mesylate, noting that NAC contains a sulfhydryl reactive group critical to its function, which distinguishes it significantly from Mitoquinone mesylate (Figure 4B). Considering that sulfhydryl-containing molecules are essential for maintaining redox homeostasis in tumor cells, we hypothesized that ponicidin-induced cell death in lung cancer cells could be mediated through depletion of intracellular sulfhydryl groups. To explore this hypothesis, we used Naph-EA-mal fluorescent dye to detect changes in sulfhydryl group content in live cells after ponicidin treatment. As expected, the results showed a significant decrease in green fluorescence intensity with increasing concentrations of ponicidin (Figure 4C), indicating depletion of intracellular sulfhydryl groups following ponicidin treatment. Co-incubation with NAC restored fluorescence intensity, suggesting recovery of intracellular sulfhydryl group content.

Furthermore, we observed a marked reduction in cellular GSH levels in lung cancer cells following ponicidin treatment compared to controls (Figure 4D). Given that GSH is a potent endogenous antioxidant, its diminished level suggests compromised intracellular antioxidant capacity. Motivated by these findings, we conducted co-incubation experiments with GSH. Consistently, supplementation with GSH restored cell morphology (Figure 4E), significantly reversed ponicidin-induced cell death in lung cancer cells, as evidenced by apoptosis analysis using flow cytometry (Figure 4F), and rescued the decline in colony formation induced by ponicidin (Figure 4G). These results suggest that alterations in sulfhydryl group content, crucial for reducing functional groups, may play a pivotal role in reversing ponicidin-induced cell death. Ponicidin induces a significant increase in ROS levels and a decrease in GSH levels in lung cancer cells, which may disrupt the intracellular redox balance and induce oxidative stress, thereby leading to cell death.

### 3.5. Ponicidin Downregulates TrxR1 in Lung Cancer Cells, a Molecule That Is Strongly Associated with Cellular Oxidative Stress

**Figure 5:**
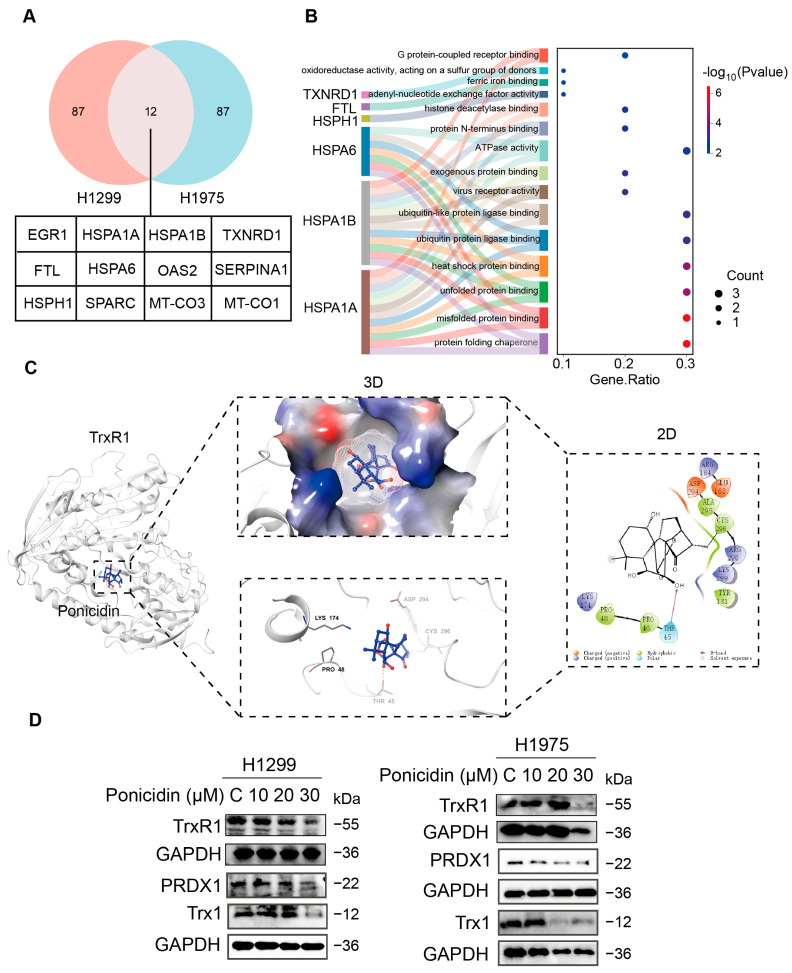
Ponicidin affects cellular oxidative stress-related pathway in lung cancer cells. (**A**) The Venn diagram illustrates 12 common differentially expressed genes between ponicidin-treated and -untreated H1299 and H1975 cells. (**B**) Pathway enrichment analyses were performed on the 12 differentially expressed genes to elucidate their biological functions. (**C**) A molecular docking study was conducted to evaluate the binding potential of ponicidin toTrxR1, identifying critical amino acids involved in this interaction. (**D**) Western blot assay showing the expression of the antioxidant proteins (PRDX1, TrxR1, and Trx1) in lung cancer cells treated with ponicidin (0, 10, 20, 30 μM) for 24 h.

In order to further elucidate the potential mechanism of action of ponicidin in lung cancer, we set up control group and ponicidin-treated group by using H1299 and H1975 cells, respectively, and cells were subjected to mRNA sequencing. To explore the common alterations of gene expression in these two lung cancer cell lines induced by ponicidin, we used venny online tool to take the intersection of the differentially expressed genes presented in the sequencing report of the two cell lines. This analysis identified 12 genes whose expression were significantly changed in ponicidin treatment group compared to control group in both H1299 and H1975 cells (Figure 5A). Pathway enrichment analysis was subsequently conducted based on the differentially expressed genes, and the top fifteen terms were visualized based on *p*-value ranking. The bubble diagram highlights the biological function of the *TXNRD1* gene, which, due to its oxidoreductase activity, is dependent on NAD(P) as an acceptor and can act on a donor containing a sulfur moiety (Figure 5B). Certain compounds can inhibit the normal activity of cytoplasmic thioredoxin (Trx1) and other substrates of the enzyme by irreversibly modifying the readily available selenocysteine residues in TrxR1 (thioredoxin reductase 1), which in turn leads to oxidative challenge, which is consistent with our previously hypothesis that ponicidin mediates cell death through depletion of sulfhydryl groups. It is therefore reasonable to speculate that TrxR1 (encoded by *TXNRD1*) may serve as a target for the antitumor activity of ponicidin.

Based on these findings, we performed molecular docking and binding free energy (ΔGbind) calculations using Covdock to evaluate the potential interaction between ponicidin and the upstream protein TrxR1. As shown in Figure 5C, ponicidin was found to bind well to the TrxR1 protein within the active cavity. Ligand covalently binds to amino acid CYS296 of the protein, and the compound forms a hydrogen-bonding interaction with THR45 residue. Then, we examined the effect of ponicidin on the level of antioxidant proteins at the protein level. The results showed that the antioxidant proteins PRDX1, Trx1, and TrxR1 were down-regulated after cells were treated with ponicidin (Figure 5D, Supplementary Figure S1), and the above results further proved the induction of oxidative stress in lung cancer cells by ponicidin. As one of the major antioxidant enzyme systems, Trx plays an important role in cellular redox control and homeostasis in cells, and TrxR, as a core regulator of the Trx system, can be inhibited pharmacologically to selectively kill cancer cells [33]. These findings collectively suggest that ponicidin may mediate oxidative stress in lung cancer cells by inhibiting TrxR1, a key upstream molecule for oxidative stress induction in lung cancer cells.

### 3.6. Ponicidin Reduces Mitochondrial Membrane Potential, Triggers ER Stress, and Induces DNA Damage in Lung Cancer Cells

**Figure 6:**
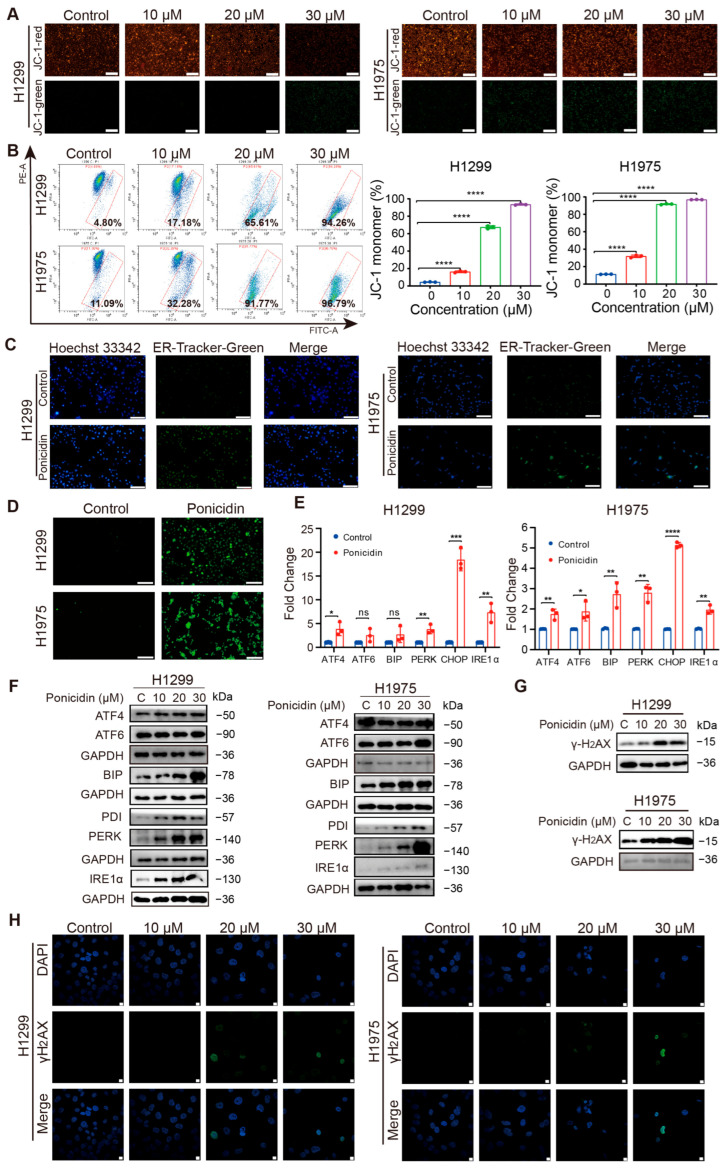
Ponicidin decreases mitochondrial membrane potential and induces endoplasmic reticulum stress and DNA damage in lung cancer cells. (**A**,**B**) H1299 and H1975 cells were treated with ponicidin for 24 h and were then stained with JC-1. Mitochondrial membrane potential levels were analyzed by fluorescence microscope, scale bar = 100 μm (**A**)) or flow cytometer (**B**) The red-gated population represents cells with depolarized mitochondria, indicated by the predominance of JC-1 monomer fluorescence; mean ± SD, *n* = 3, **** *p* < 0.0001. (**C**) H1299 and H1975 cells were treated with ponicidin for 24 h. The cells were then stained with ER-Tracker Green, and the intensity of green fluorescence in H1299 and H1975 cells is used to indicate whether endoplasmic reticulum dilation has occurred, scale bar = 200 μm. (**D**) The calcium indicator Fluo-4 AM detects intracellular Ca^2+^ levels in H1299 and H1975 cells, scale bar = 200 μm. (**E**) ER stress-related genes was detected using RT-qPCR in H1299 and H1975 cells with or without ponicidin treatment, mean ± SD, *n* = 3, * *p* < 0.05, ** *p* < 0.01, *** *p* < 0.001, **** *p* < 0.0001, ns: no significance. (**F**) ER stress-related proteins were analyzed using Western blot in H1299 and H1975 cells with or without ponicidin treatment. (**G**) H1299 and H1975 cells were treated with ponicidin at various concentrations, the expression of γ-H_2_AX was detected by Western blot analysis. (**H**) γ-H_2_AX expression in H1299 and H1975 cells with or without ponicidin (30 μM) treatment was analyzed by immunofluorescence (green). Nuclei were counterstained with DAPI (blue), scale bar = 10 μm.

The elevation of ROS levels induced by ponicidin can have diverse effects on various organelles, notably mitochondria and the ER. To investigate these effects, we utilized JC-1, a fluorescent probe commonly used to assess mitochondrial membrane potential. According to our findings, as ponicidin concentrations increased, red fluorescence gradually decreased and green fluorescence increased (Figure 6A), indicating a reduction in mitochondrial membrane potential in lung cancer cells treated with ponicidin. This observation was further validated by flow cytometry, showing consistent observation that ponicidin treatment induced the decrease in mitochondrial membrane potential in lung cancer cells (Figure 6B).

Additionally, the ER is essential for both quality control and protein production. When ER homeostasis is disturbed, ER stress can result, which is indicated by the buildup of misfolded or unfolded proteins in the ER lumen [34]. Given the significant impact of oxidative stress on cellular processes, it was reasonable to hypothesize that ER stress might accompany oxidative stress induced by ponicidin. Using ER-Tracker Green, a fluorescent probe selective for the ER, we observed a notable increase in fluorescence intensity after 24 h of ponicidin treatment in H1299 and H1975 cells (Figure 6C), indicating ER expansion, swelling, and vacuolation. Since the ER is a major reservoir of calcium ions in cells, and disruptions in ER function can lead to dysregulation of cellular calcium homeostasis and subsequent ER stress, we then detected the changes of calcium ions in lung cancer cells after ponicidin treatment. Using Fluo-4 AM, a calcium ion fluorescent probe, we observed increased green fluorescence in lung cancer cells treated with ponicidin, indicating elevated levels of free calcium ions (Figure 6D). Additionally, ponicidin treatment significantly upregulated the expression of ER stress-related genes such as ATF4, PERK, CHOP, and IRE1α (Figure 6E). The elevation of important ER stress proteins BIP, PDI, PERK, and IRE1α at the protein level in lung cancer cells treated with increasing concentrations of ponicidin supported this rise in gene expression (Figure 6F, Supplementary Figure S2).

Additionally, increased ROS levels are known to contribute to DNA damage [35]. Therefore, we assessed the effect of ponicidin on intracellular levels of γ-H_2_AX, a marker of DNA damage, using Western blotting. Our results demonstrated a gradual increase in γ-H_2_AX levels with increasing ponicidin concentration in both H1299 and H1975 cells (Figure 6G, Supplementary Figure S3), indicating induction of DNA damage by ponicidin treatment. Immunofluorescence assays further confirmed DNA damage induction, as evidenced by the emergence and intensification of green fluorescence in ponicidin-treated lung cancer cells (Figure 6H). These findings collectively illustrate that ponicidin induces mitochondrial dysfunction, ER stress, and DNA damage in lung cancer cells. The observed effects underscore the complex interplay between oxidative stress, organelle dysfunction, and cellular responses to ponicidin treatment in lung cancer cells.

### 3.7. NAC Attenuates Ponicidin-Induced Organellar Stress and DNA Damage in Lung Cancer Cells

**Figure 7:**
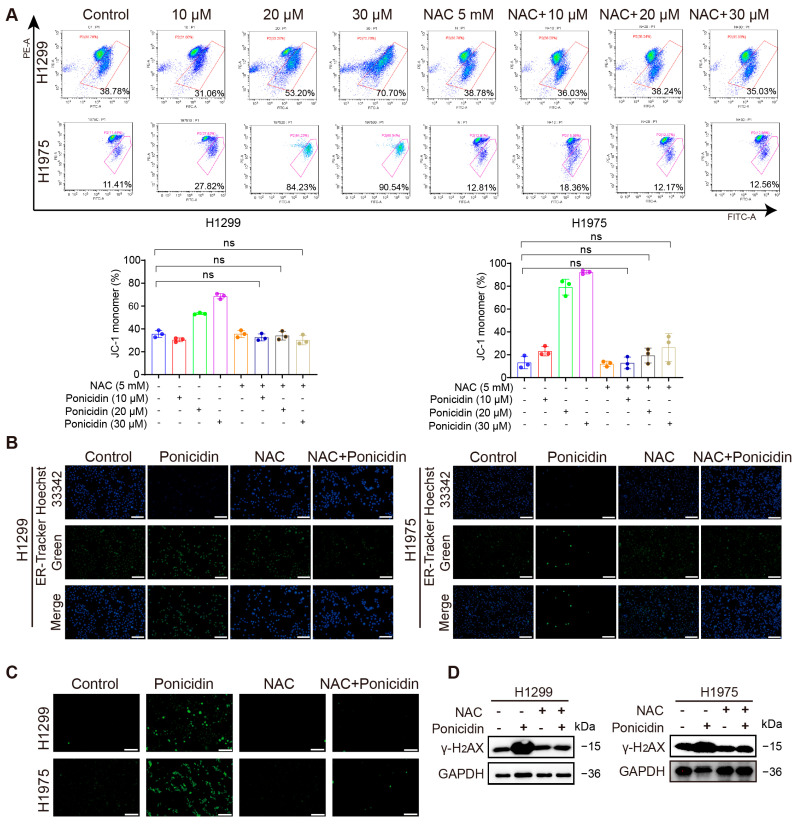
NAC antagonizes the effects of ponicidin on mitochondria, ER, and DNA damage in lung cancer cells. (**A**) The impact of ponicidin in combination with NAC on the mitochondrial membrane potential of lung cancer cells was evaluated using flow cytometry; The red-gated population represents cells with depolarized mitochondria, indicated by the predominance of JC-1 monomer fluorescence; mean ± SD, *n* = 3, ns: no significance; “-”: without the corresponding treatment; “+”: with the corresponding treatment. (**B**) The ER-Tracker Green Kit was utilized to assess the impact of ponicidin in combination with NAC on the endoplasmic reticulum of lung cancer cells, scale bar = 200 μm. (**C**) The Fluo-4 Ca^2+^ Kit was employed to investigate the effects of ponicidin in combination with NAC on the endoplasmic reticulum of lung cancer cells, scale bar = 200 μm. (**D**) H1299 and H1975 cells were treated with ponicidin, with or without co-treatment with NAC. The expression of γ-H_2_AX was subsequently assessed by Western blot analysis.

To elucidate the potential link between ROS and the organellar damage induced by ponicidin, we further used NAC to assess whether ROS elevation contributes to mitochondrial impairment, ER stress, and DNA damage in lung cancer cells. As shown in Figure 7A, pretreatment with NAC effectively restored mitochondrial membrane potential in cells exposed to ponicidin, indicating that ROS accumulation plays a role in ponicidin-induced mitochondrial dysfunction. Furthermore, NAC co-treatment markedly reduced both ER-Tracker green fluorescence and intracellular Ca^2+^ signals (Figure 7B,C), suggesting attenuated ER dilatation and lowered ER calcium load compared to ponicidin treatment alone. Consistent with these findings, Western blot analysis demonstrated that NAC substantially suppressed the upregulation of the DNA damage marker γ-H_2_AX induced by ponicidin (Figure 7D, Supplementary Figure S4). These results suggest that the mitochondrial damage, ER stress, and DNA damage observed following ponicidin treatment are associated with the concurrent increase in cellular ROS levels, as these effects were significantly attenuated by the ROS scavenger NAC.

### 3.8. Ponicidin Inhibits Lung Cancer Cell Growth by Regulating Oxidative Stress In Vivo

**Figure 8:**
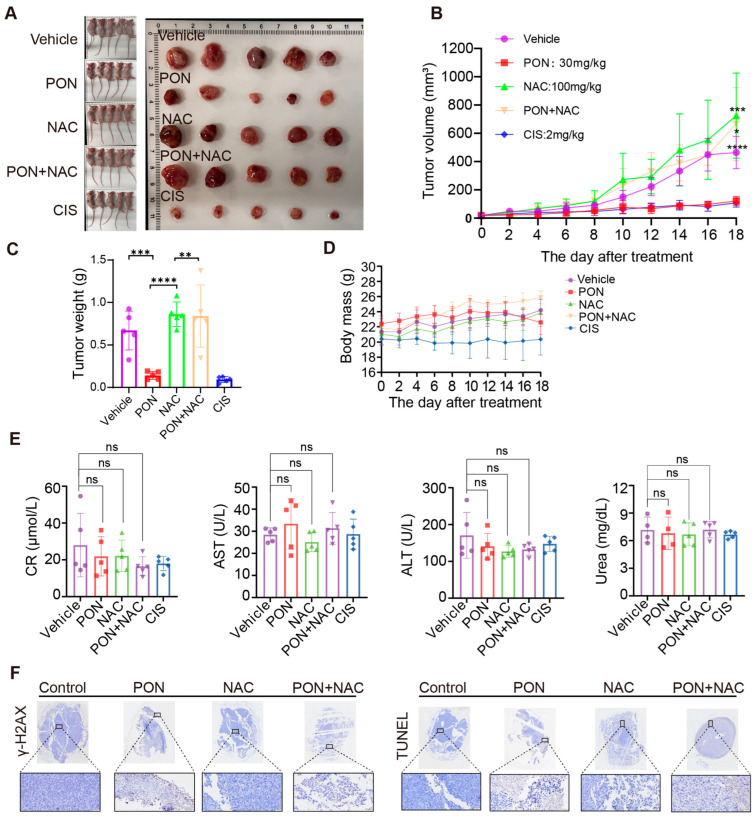
NAC attenuates the inhibitory effect of ponicidin on the growth of lung cancer cells in vivo. (**A**) Mice were euthanized after 18 days’ treatment, and tumors were extracted and photographed. (**B**–**D**) Tumor volumes, weights and body mass were measured and monitored across different treatment groups. Data are presented as mean ± SD, with *n* = 5, *: *p* < 0.05, **: *p* < 0.01, ***: *p* < 0.001, ****: *p* < 0.0001. (**E**) Blood samples were collected for biochemical analysis, mean  ±  SD, *n* = 4–5, ns: no significance. (**F**) Immunohistochemical analysis of γ-H_2_AX expression and TUNEL staining were employed to evaluate tumor cell DNA damage and apoptosis, respectively. The boxed area below shows an enlarged view, scale bar = 50 μm.

In order to properly test the theory that ponicidin prevents lung cancer by causing oxidative stress in the in vivo setting, a subcutaneous tumor model was created in nude mice using H1299 cells, and the impact of NAC on ponicidin’s in vivo effectiveness was evaluated. The mice were divided into five groups at random: control group, ponicidin treatment group (30 mg/kg), NAC group (100 mg/kg), ponicidin + NAC group, and cisplatin positive control group with 2 mg/kg. The addition of NAC considerably counteracted the inhibitory effects of ponicidin on lung cancer cells, and the tumor volume and weights of the ponicidin-treated group were significantly less than those of the control group, which is consistent with the findings of the in vitro experiments (Figure 8A–C). Additionally, ponicidin shows comparable anti-lung tumor effects with cisplatin, demonstrating its potential in the development of anti-lung cancer drugs. Importantly, body mass (Figure 8D) and blood biochemical assays from each xenograft mouse (Figure 8E) indicated that ponicidin did not exhibit significant toxicity or adverse effects compared with both the untreated and the positive control groups. Additional tissue immunofluorescence labeling revealed that tumors from the ponicidin-treated group had significantly higher levels of the DNA damage marker γ-H_2_AX, along with an increase in TUNEL staining to mark apoptotic cells (Figure 8F). These effects were less pronounced in the treatment groups receiving ponicidin combined with NAC. Taken together, these results provide compelling evidence that ponicidin suppresses the proliferation of lung cancer cells by controlling oxidative stress and suggest that it has great potential for use as an anti-lung cancer medication.

## 4. Discussion

Lung cancer is a serious health concern [36], necessitating the exploration of novel therapeutic approaches. Natural products derived from microorganisms, plants, and animals are known to have bioactive chemicals that may be used in cancer treatment [37]. *Rabdosia rubescens*, a traditional Chinese medicinal herb, contains ponicidin as one of its active constituents [38]. Ponicidin has shown anti-tumor properties against a variety of cancer types, including colorectal cancer [39], gallbladder cancer [24], pancreatic cancer [40], and gastric cancer [41], yet its impact on lung cancer cells and underlying molecular mechanisms remain underexplored. According to our research, ponicidin efficiently causes lung cancer cells to die while posing little harm to healthy lung cells. This suggests that ponicidin is a promising therapy option for lung cancer and should be further studied for clinical translation.

Cellular redox homeostasis is pivotal in regulating the equilibrium between oxidative and antioxidative reactions within cells, influencing various biological processes [42]. The oxidative stress status within cells is primarily governed by the levels of ROS [43]. When intracellular ROS levels exceed antioxidant defense mechanisms, such as those mediated by GSH, cancer cells become vulnerable to various forms of cell death [44,45]. Modulating tumor redox homeostasis with compounds that regulate ROS levels presents a promising strategy for selective cancer cell eradication [46,47,48,49]. Our investigation demonstrated that ponicidin induces elevated ROS levels in lung cancer cells, indicating its potential to induce oxidative stress. Subsequent experiments revealed that the ROS scavenger NAC effectively reversed ponicidin-induced cell death in lung cancer cells, whereas Mitoquinone mesylate (MitoQ) did not. The observed increase in total cellular ROS, detected using the broad-spectrum probe DCFH-DA. The differential rescue by NAC but not MitoQ suggests that the pivotal cytotoxic ROS induced by ponicidin may originate from sources beyond the mitochondria or involve ROS species not effectively neutralized by mitochondrial-targeted scavenging. Furthermore, analysis showed a significant reduction in intracellular sulfhydryl groups and glutathione levels in ponicidin-treated lung cancer cells, indicating disruption of redox balance. These findings were supported by the decreased expression of antioxidant-related proteins such as PRDX1, TrxR1 and Trx1 in ponicidin-treated lung cancer cells, underscoring ponicidin’s mechanism of inducing cell death through modulation of intracellular oxidative stress. TrxR, as a key component of the thioredoxin system, is usually up-regulated in cancers and is considered as a target for anticancer drug development [50,51]. As a regulator of cellular redox status, an imbalance in the TrxR/Trx system affects other antioxidant factors associated with it [52], and oxidative disturbances are often accompanied by increased HSP synthesis [53]. This is consistent with the observation in our study that ponicidin significantly upregulated HSP family members while downregulating TrxR1 in lung cancer cells. Subsequent computational molecular docking analysis predicted a potential interaction between ponicidin and TrxR1, suggesting that ponicidin might bind to this key antioxidant enzyme. This in silico prediction, combined with the observed downregulation of TrxR1 and the associated oxidative stress phenotype, forms a hypothesis that ponicidin could act, in part, by modulating the TrxR1/Trx system. This potential mechanism warrants further experimental investigation, such as through direct binding assays and enzymatic activity studies, to definitively establish whether ponicidin functions as a TrxR1 inhibitor. In addition, in preliminary investigations promoted by this study, qPCR analysis suggested an upward trend in the expression of DUOX2, a ROS-generating dual oxidase, in ponicidin-treated H1975 cells (Supplementary Figure S5). While this observation requires further validation and functional exploration, it hints at the possibility that ponicidin may modulate specific upstream ROS sources, such as DUOX2, to initiate or amplify the oxidative cascade. Future studies are warranted to systematically profile NOX/DUOX family members and define their contribution to ponicidin’s mechanism of action.

The ER is essential for calcium storage and protein folding, both of which are necessary for preserving cellular homeostasis. Oxidative stress can disrupt ER function [54], leading to ER stress. Our investigations revealed that ponicidin treatment expanded the ER and increased free calcium ion levels in lung cancer cells. Additionally, there was elevated expression of key ER stress markers such as BIP, PERK, and IRE1α, indicating the induction of ER stress by ponicidin. In addition, given that oxidative stress is a primary contributor to DNA damage, our findings also demonstrated elevated levels of γ-H_2_AX, a recognized marker of DNA damage, in ponicidin-treated lung cancer cells. This observation suggests that ponicidin induces DNA damage as part of its cytotoxic mechanism in lung cancer cells.

Furthermore, in vivo research has demonstrated that ponicidin dramatically slows the growth of tumors in mice with lung cancer. Along with decreasing tumor weight and volume, ponicidin increased the number of TUNEL-positive cells and the expression level of the DNA damage marker γ-H_2_AX. In the in vivo setting, the effects of ponicidin on lung cancer cell growth were largely counteracted by the ROS scavenger NAC, suggesting that ponicidin modulates the onset of oxidative stress, thereby inducing lung cancer cell death. Importantly, ponicidin was not significantly toxic to mice, as confirmed by H&E staining of various tissues and serum indices used to assess the metabolic function of the animals, holding promise as a potential therapeutic agent for lung cancer.

## 5. Conclusions

In conclusion, this study elucidates that ponicidin induces cell death and disrupts redox homeostasis in lung cancer cells, primarily by depleting sulfhydryl antioxidants, including glutathione, and downregulating key antioxidant proteins such as Trx1, TrxR1, and Prdx1. TrxR1 was computationally predicted as a potential target of ponicidin in oxidative stress modulation, offering a mechanistic hypothesis for its antitumor activity. Furthermore, ponicidin triggers ER stress, impairs mitochondrial function, perturbs calcium homeostasis, and induces DNA damage in lung cancer cells. In vivo studies reveal significant antitumor efficacy and a favorable safety profile of ponicidin in a xenograft model. These findings highlight ponicidin as a promising preclinical candidate worthy of further investigation for lung cancer therapy.

## Figures and Tables

**Table 1:** Primers sequence for RT-qPCR.

Gene	Accession Number	Forward Primer	Reverse Primer
*BIP*	ID: 3309	CATCACGCCGTCCTATGTCG	CGTCAAAGACCGTGTTCTCG
*CHOP*	ID: 1649	GGAAACAGAGTGGTCATTCCC	CTGCTTGAGCCGTTCATTCTC
*PERK*	ID: 9451	TGTCGCCAATGGGATAGTGACGAA	AATCCGGCTCTCGTTTCCATGTCT
*ATF4*	ID: 468	CTCCGGGACAGATTGGATGTT	GGCTGCTTATTAGTCTCCTGGAC
*ATF6*	ID: 22926	TCCTCGGTCAGTGGACTCTTA	CTTGGGCTGAATTGAAGGTTTTG
*GAPDH*	ID: 2597	GGAGCGAGATCCCTCCAAAAT	GGCTGTTGTCATACTTCTCATGG
*IRE1α*	ID: 2081	CACAGTGACGCTTCCTGAAAC	GCCATCATTAGGATCTGGGAGA

## Data Availability

The original contributions presented in this study are included in the article. Further inquiries can be directed to the corresponding authors.

## Supplementary Materials

The following supporting information can be downloaded at: https://www.mdpi.com/article/10.3390/antiox15010100/s1. Figure S1. Original Western blot membranes corresponding to Figure 5D and the relative protein expression levels quantified by densitometric analysis of Western blots and normalized to GAPDH. The grayscale values are annotated in red on the respective bands. Figure S2. Original Western blot membranes corresponding to Figure 6F and the relative protein expression levels quantified by densitometric analysis of Western blots and normalized to GAPDH. The grayscale values are annotated in red on the respective bands. Figure S3. Original Western blot membranes corresponding to Figure 6G and the relative protein expression levels quantified by densitometric analysis of Western blots and normalized to GAPDH. The grayscale values are annotated in red on the respective bands. Figure S4. Original Western blot membranes corresponding to Figure 7D and the relative protein expression levels quantified by densitometric analysis of Western blots and normalized to GAPDH. The grayscale values are annotated in red on the respective bands. Figure S5. Detection of NOX2, NOX4, DUOX1, and DUOX2 in H1975 cells after treatment with different concentrations of ponicidin.
