# Supplementary material for: Ponicidin Inhibits Lung Cancer Progression Through Coordinated Downregulation of Sulfhydryl Antioxidants and TrxR1
Source: Antioxidants (Basel). 2026 Jan 13;15(1):100. doi: 10.3390/antiox15010100 (PMC12837182; doi:10.3390/antiox15010100)
Supplement: Supplementary file 1 [file antioxidants-15-00100-s001.zip › antioxidants-4060956-supplementary.pdf]

Ponicidin Inhibits Lung Cancer Progression Through Coordinated Downregulation of Sulfhydryl Antioxidants and TrxR1

Yufei Huang[1,#], Yanfen Liu[1,#], Zehua Liao[1], Ruonan Zhang[1], Xinbing Sui[1] and Xueni Sun[1,*]

1 School of Pharmacy, Hangzhou Normal University, Hangzhou, Zhejiang 311121, China

* Correspondence: xnsun@hznu.edu.cn (Xueni Sun)

# These authors contributed equally to this work as first authors.

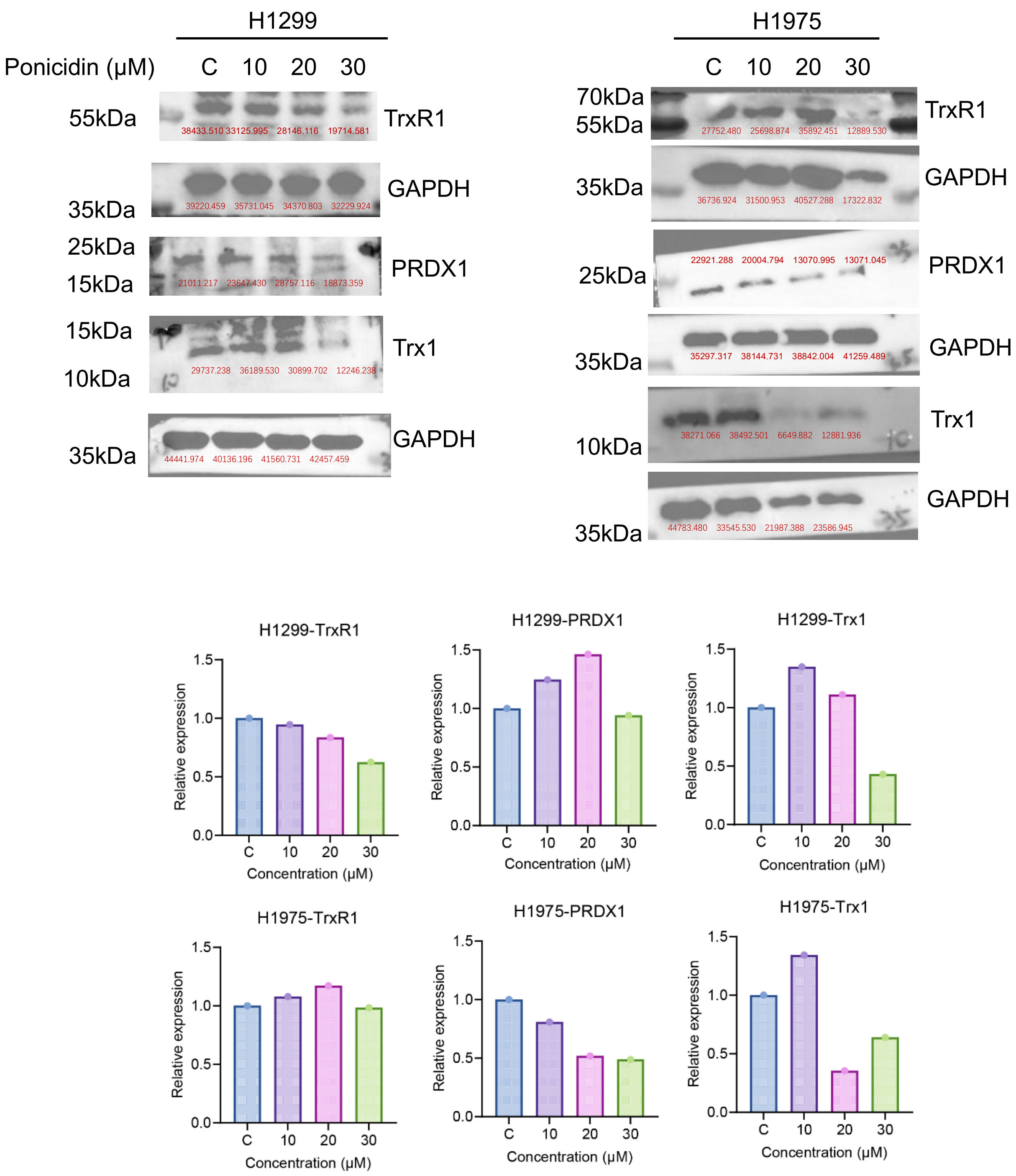

Figure S1. Original Western blot membranes corresponding to Figure 5D and the relative protein expression levels quantified by densitometric analysis of Western blots and normalized to GAPDH. The grayscale values are annotated in red on the respective bands.

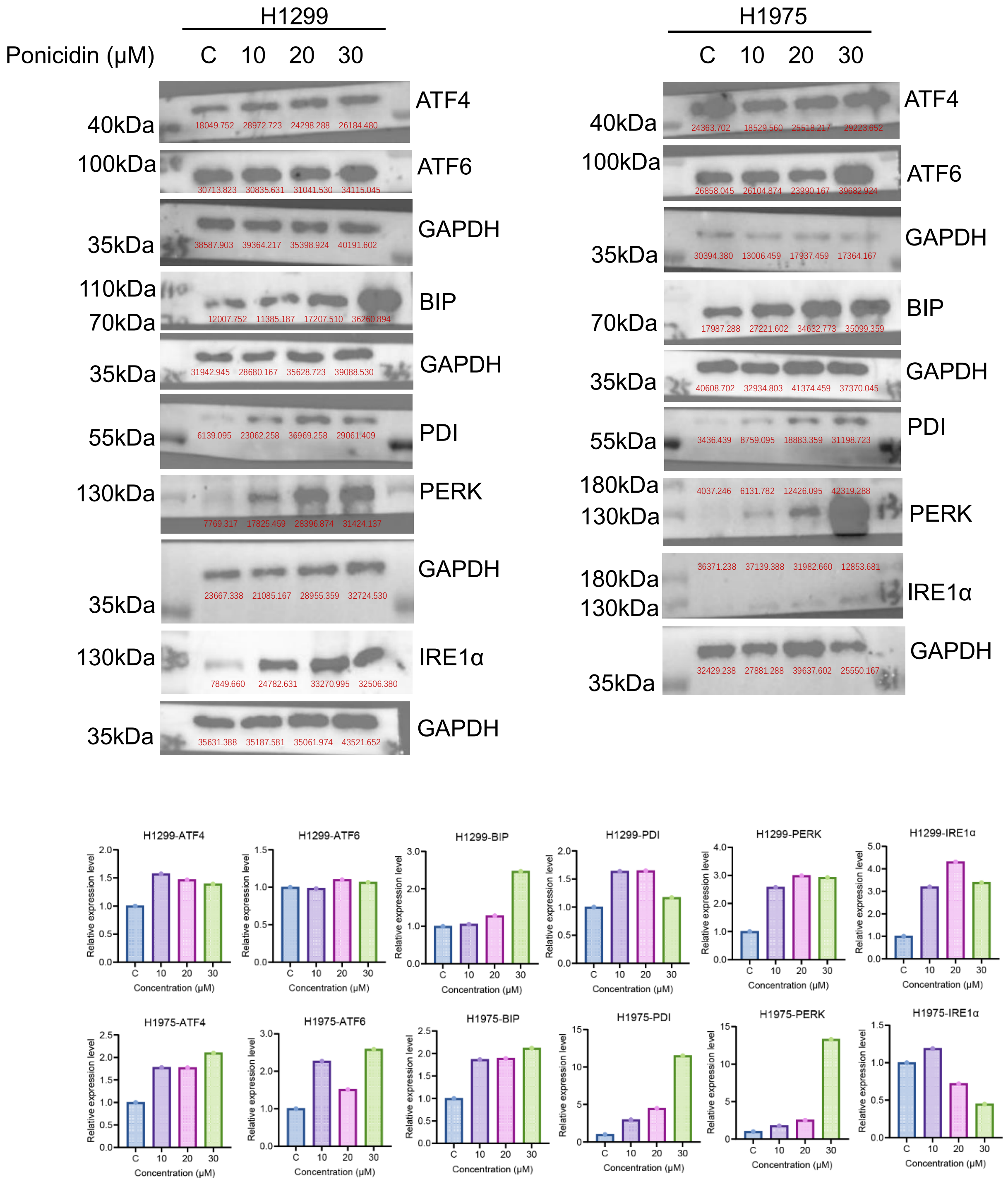

Figure S2. Original Western blot membranes corresponding to Figure 6F and the relative protein expression levels quantified by densitometric analysis of Western blots and normalized to GAPDH. The grayscale values are annotated in red on the respective bands.

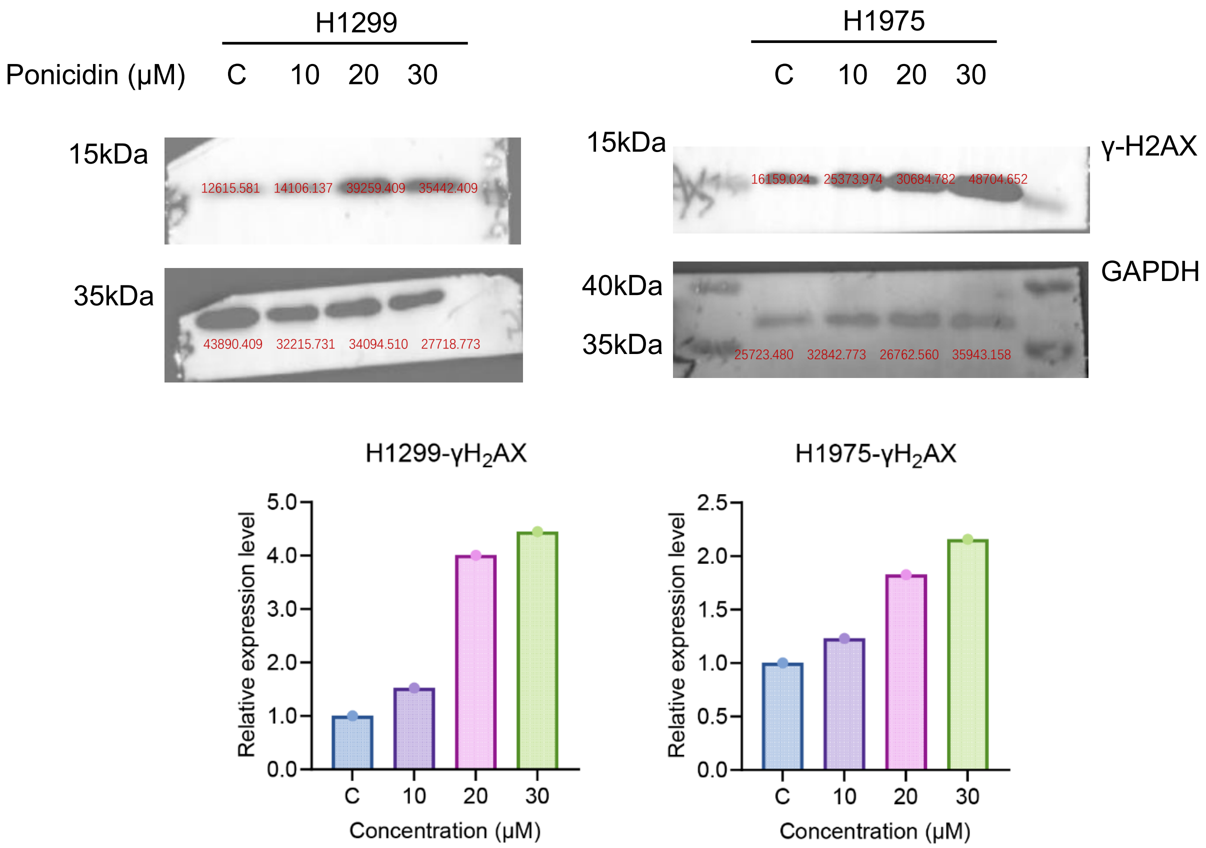

Figure S3. Original Western blot membranes corresponding to Figure 6G and the relative protein expression levels quantified by densitometric analysis of Western blots and normalized to GAPDH. The grayscale values are annotated in red on the respective bands.

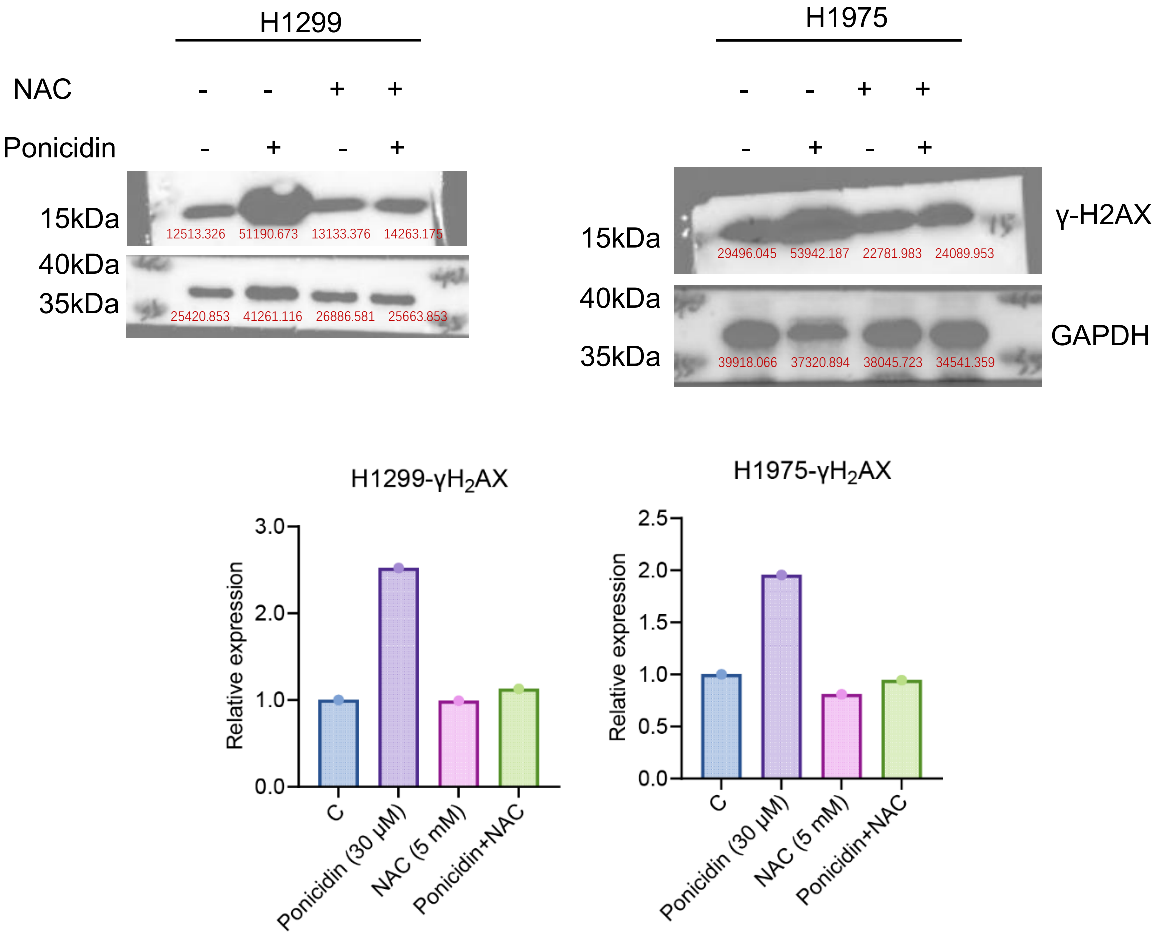

Figure S4. Original Western blot membranes corresponding to Figure 7D and the relative protein expression levels quantified by densitometric analysis of Western blots and normalized to GAPDH. The grayscale values are annotated in red on the respective bands.

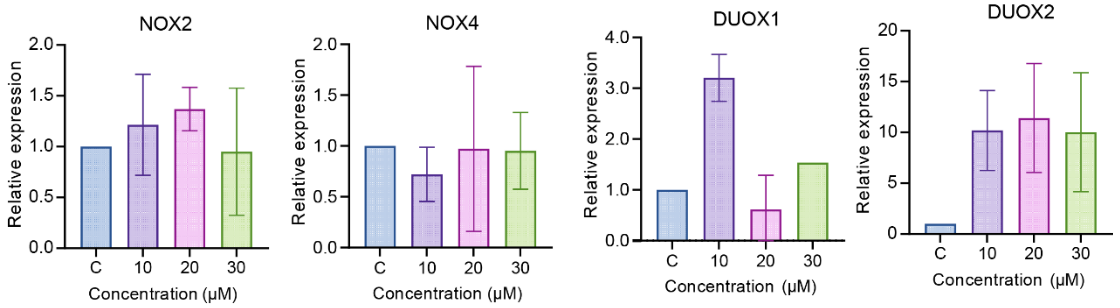

Figure S5. Detection of NOX2, NOX4, DUOX1, and DUOX2 in H1975 cells after treatment with different concentrations of ponicidin.
